# Examination of the relationship between autistic traits and psychotic experiences: a cross-sectional study among community young adults

**DOI:** 10.1192/j.eurpsy.2026.10996

**Published:** 2026-07-31

**Authors:** L. S. Chaibi, A. Kammoun, W. Cherif, M. Cheour, F. Fekih-Romdhane

**Affiliations:** 1 Faculty of Medecine of Tunis; 2 Psychiatry; 3 Ibn Omran, Razi Hospital, Tunis, Tunisia

## Abstract

**Introduction:**

Autistic traits (ATs) and psychotic experiences (PEs) were often viewed as distinct phenomena, but growing evidence has suggested that both exist on a continuum within the general population. Despite this, research on the co-occurrence of subthreshold symptoms remains limited, particularly in non-clinical populations.

**Objectives:**

This study aimed to investigate the relationship between ATs and PEs in a large sample of young adults from the general population and to investigate the mediating and moderating variables that may influence this relationship (i.e., Social Pain, loneliness, Alexythymia, childhood trauma).

**Methods:**

This cross-sectional study was conducted from February 1st to April 30, 2024.. Data were collected using self-administered questionnaires, including validated Arabic versions of the Autism-Spectrum Quotient, Prodromal Questionnaire-Brief, Social Pain Questionnaire, and Jong-Gierveld Loneliness Scale. Mediation analyses were conducted to explore the indirect effects of social pain and loneliness on the association between ATs and PEs.

**Results:**

The total of partcipants was 7,646 participants aged 18 to 35 (mean age 22.55 ± 4.00 years). Mediation analysis revealed that both loneliness (indirect effect: Beta = .19; Boot SE = .02; Boot CI .16, .22) and social pain (indirect effect: Beta = .04; Boot SE = .01; Boot CI .02, .06) partially mediated the relationship between ATs and PEs. Higher ATs were associated with increased loneliness and social pain, both of which were significantly linked to more severe PEs. Sociodemographic factors, including sex, education level, and smoking status, were also found to be significantly correlated with higher PQ-B scores.

**Conclusions:**

This research has contributed to a better understanding of the complex interplay between ATs and PEs and highlights the importance of addressing these factors for mental health interventions.This study has provided new insights into the mediating roles of social pain and loneliness in the relationship between Ats and PEs.

**Disclosure of Interest:**

None Declared

